# ICTV Virus Taxonomy Profile: Polymycoviridae 2022

**DOI:** 10.1099/jgv.0.001747

**Published:** 2022-05-31

**Authors:** Ioly Kotta-Loizou, Robert H. A. Coutts, ICTV Report Consortium

**Affiliations:** 1 Department of Life Sciences, Faculty of Natural Sciences, Imperial College London, London SW7 2AZ, UK; 2 Department of Clinical, Pharmaceutical and Biological Sciences, School of Life and Medical Sciences, University of Hertfordshire, Hatfield AL10 9AB, UK

**Keywords:** *Polymycoviridae*, *Polymycovirus*, taxonomy, ICTV report

## Abstract

Members of the family *Polymycoviridae* are small viruses with multi-segmented and non-conventionally encapsidated double-stranded (ds) RNA genomes. Typically, polymycoviruses have four genomic segments, although some have up to eight. The genus *Polymycovirus* includes several species whose members infect fungi (ascomycetes and basidiomycetes), and oomycetes, altering host morphology, sporulation, growth and virulence. This is a summary of the International Committee on Taxonomy of Viruses (ICTV) Report on the family *Polymycoviridae,* which is available at ictv.global/report/polymycoviridae.

## Virion

**Fig. 1.**
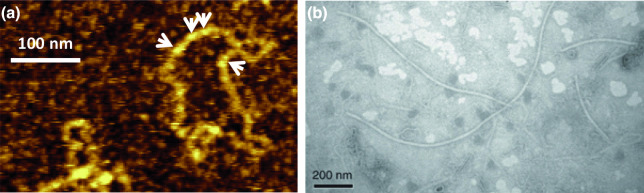
Polymycovirus virions. (a) Atomic force microscopy of purified Aspergillus fumigatus tetramycovirus 1 non-conventional virions; white arrows indicate the viral protein coating the dsRNA genome. (Adapted from Ref. [1]). (b) Transmission electron microscopy of purified Colletotrichum camelliae filamentous virus 1 conventional virions. (Adapted from Ref. [4] under CC BY 4.0).

 Most polymycoviruses form non-conventional virions, whereby genomic dsRNA is coated by a viral protein (Table 1) [1,3]. Only Colletotrichum camelliae filamentous virus 1 is believed to have a filamentous capsid [4], 10–20 nm in width and >1000 nm in length (Fig. 1).

**Table 1.** Characteristics of members of the family *Polymycoviridae*

Example	Aspergillus fumigatus tetramycovirus 1 (dsRNA 1: HG975302; dsRNA 2: HG975303; dsRNA 3: HG975304; dsRNA 4: HG975303), species *Aspergillus fumigatus polymycovirus 1*, genus *Polymycovirus*
**Virion**	Non-conventionally encapsidated dsRNA, coated with viral protein
**Genome**	A total of 7.5–12.5 kbp of dsRNA in a multipartite genome (usually four segments, up to eight)
**Replication**	Both dsRNA and ssRNA can be isolated from infected fungal hosts. Virions accumulate in the cytoplasm
**Translation**	From positive-sense transcripts of genomic dsRNAs
**Host range**	Fungi (ascomycetes and basidiomycetes), oomycetes
**Taxonomy**	Realm *Riboviria*; the genus *Polymycovirus* includes >9 species

## Genome

**Fig. 2.**
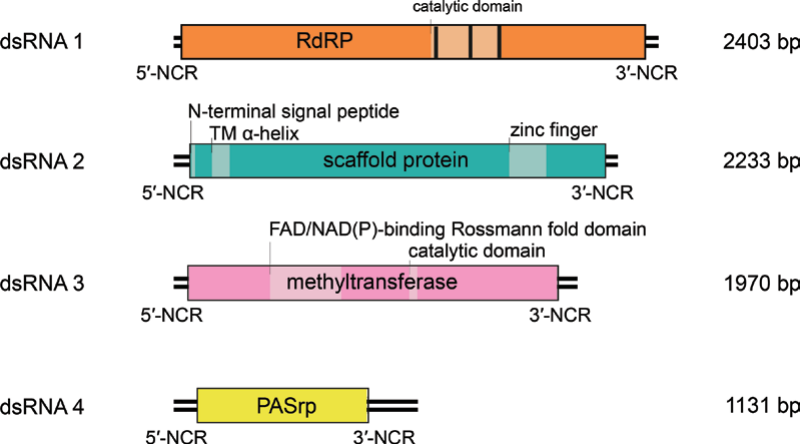
Genome organization of Aspergillus fumigatus tetramycovirus 1. ORFs are represented by rectangular boxes with internal light-coloured boxes representing known and/or predicted motifs and domains, such as the RdRP_1 motif (PF00680) in dsRNA 1 and Methyltransf_25 (PF13649) in dsRNA 3. Vertical lines indicate conserved RdRP motifs.

Polymycovirus genomes range from 7.5–12.5 kbp and comprise four to eight dsRNA segments. Each segment contains a single open reading frame (ORF) flanked by long non-coding regions (NCRs) with conserved termini (Fig. 2).

The ORF of dsRNA 1 encodes an RNA-directed RNA polymerase (RdRP) belonging to the protein family RdRP_1 (Pfam PF00680) and has three conserved motifs. The ORF of dsRNA 2 encodes a protein of unknown function containing a conserved N-terminus and a cysteine-rich, zinc finger-like motif. The ORF of dsRNA 3 encodes a methyl transferase, responsible for adding a capping structure to the 5′-termini of the positive-sense strands of viral dsRNAs [1,2]. The ORF of dsRNA 4 encodes a proline–alanine–serine-rich protein (PASrp). When present, dsRNAs 5–8 encode proteins of unknown function that are non-homologous between different viruses.

## Replication

Polymycoviruses were the first dsRNA viruses found to be infectious not only as purified entities but also as naked dsRNA [1,3, 4]. Replication has not been characterized in detail. Virions accumulate in the cytoplasm.

## Pathogenicity

Polymycovirus infection has been associated with various host alterations, including changes in pigmentation [1,2, 4, 5], sectoring [1,2], decreased host growth [4] and host virulence [4], as well as increased sporulation [5], host growth [2,5] and host virulence [2]. Infection has also been reported to increase sensitivity to antifungals [3] and to the bacterium *Pseudomonas aeruginosa* [6]. Although the molecular mechanisms underpinning the above phenotypes have not been elucidated, there is evidence that polymycoviruses modulate host carbon, nitrogen and iron metabolism [5,7]. Finally, polymycoviruses are targeted by the host RNA silencing machinery [8].

## Taxonomy

Current taxonomy: ictv.global/taxonomy. Viruses in the family *Polymycoviridae* are most closely related to Hadaka virus 1 isolates, positive-sense (+) single-stranded (ss) RNA viruses in the family *Hadakaviridae*. Polymycovirus-encoded RdRPs are also related to those of (+)ssRNA viruses in the families *Astroviridae*, *Caliciviridae* and *Picornaviridae*, and of dsRNA viruses in the family *Partitiviridae* in the phylum *Pisuviricota*. The GDNQ motif, typically found in the RdRP of negative-sense ssRNA viruses of the order *Mononegavirales*, is conserved in all members of the family *Polymycoviridae*, instead of the GDD motif found in most dsRNA and (+)ssRNA viruses. Polymycoviruses appear to be intermediate between dsRNA and (+)ssRNA viruses, as well as between encapsidated and capsidless RNA viruses [1].

## Resources

Full ICTV Report on the family *Polymycoviridae*: ictv.global/report/polymycoviridae.

## Abbreviations

(+): positive-sense
ds: double-stranded
NCR: non-coding region
ORF: open reading frame
PASrp: proline-alanine-serine-rich protein
RdRP: RNA-directed RNA polymerase
